# A comprehensive custom panel evaluation for routine hereditary cancer testing: improving the yield of germline mutation detection

**DOI:** 10.1186/s12967-020-02391-z

**Published:** 2020-06-10

**Authors:** Carolina Velázquez, Enrique Lastra, Francisco Avila Cobos, Luis Abella, Virginia de la Cruz, Blanca Ascensión Hernando, Lara Hernández, Noemí Martínez, Mar Infante, Mercedes Durán

**Affiliations:** 1 Cancer Genetics Group, Institute of Genetics and Molecular Biology (UVa-CSIC), Sanz y Forés 3, 47003 Valladolid, Spain; 2 Unit of Genetic Counseling in Cancer, Complejo Hospitalario de Burgos, Burgos, Spain; 3 Center for Medical Genetics Ghent (CMGG), Ghent University, 9000 Ghent, Belgium; 4 Unit of Genetic Counseling in Cancer, Hospital Universitario Rio Hortega, Valladolid, Spain; 5 IRCM, Institut de Recherche en Cancérologie de Montpellier, INSERM U1194, Université de Montpellier, Montpellier, France

**Keywords:** Germline mutation, Genetic counselling, Hereditary Cancer syndrome, On-Demand gene panel, Next generation sequencing

## Abstract

**Background:**

In the context of our Regional Program of Hereditary Cancer, individuals fulfilling the criteria are tested for germline mutations to subsequently establish the clinical management. Our standard diagnostic approach focuses on sequencing a few classic high-risk genes, a method that frequently renders uninformative genetic results. This study aims to examine the improved yield offered by an On-Demand panel.

**Methods:**

We designed an On-Demand panel for the analysis of 35-genes associated with inherited cancer susceptibility in a total of 128 cases of Hereditary Breast and Ovarian Cancer (HBOC) and Hereditary Nonpolyposis Colorectal Cancer (HNPCC).

**Results:**

Eighteen deleterious mutations were detected, in both routinely (*BRCA2, MLH1, MSH2, PMS2*) and non-routinely (*ATM, BLM, BRIP1, CHEK2, MUTYH*) tested genes. The screening extended to 35 genes rendered by patients carrying several- up to 6-Variants of Unknown Significance (VUS). Moreover, we confirmed the splicing disruption at RNA level for a not previously reported *BRIP1* splicing mutation. Using an On-Demand panel, we identified 18 pathogenic mutation carriers, seven of which would have gone unnoticed with traditional analysis.

**Conclusions:**

Our results reinforce the utility of NGS gene panels in the diagnostic routine to increase the performance of genetic testing, especially in individuals from families with overlapping cancer phenotypes.

## Background

Carriers of germline mutations in cancer susceptibility genes represent a small but relevant proportion of all cancer cases. This is because an effective clinical management can be implemented in the families when the genetic predisposition has been identified [1]. Traditional genetic screening is based on the analysis of classical high penetrance genes that only explain the genetic predisposition in a reduced number of families [2]. Moreover, the process is time-consuming and expensive, underscoring the unquestionable need not only to increase the number of analyzed genes, but also to do so by a scalable method. In that regard, the development of gene cancer panels, an application of massively parallel sequencing technology, is replacing sequential single-gene testing. Using gene-panels, it is possible to explore several genes at once, increasing the chance of finding the causal mutation. Moreover, gene-panels represent an affordable next generation sequencing (NGS) application for clinical practice. A critical decision regarding the gene-panel implementation is to look into the right genes for the particular hereditary syndrome. To satisfy this demand, customized panels have started to be offered.

These customized panels are being used in a large number of diagnostic laboratories, providing the opportunity to discuss the implementation process and increased mutation yields [3–5]. In general, the cohort of cancer cases can meet the criteria for different syndromes due to the overlapping phenotypes. On a practical level, the panel should include the candidate genes that match with the phenotype of the samples. A thorough analysis of the results is essential for evaluating the implementation of these custom panels and to optimize their use [6].

We aim to incorporate the study of an On-Demand gene panel related to HBOC (Hereditary Breast and Ovarian Cancer) and HNPCC (Hereditary Non Polyposis Colon Cancer) in the clinical routine of one of the reference laboratories in the Hereditary Community Cancer Program of Castile & Leon.

## Materials and methods

### Patients

A total of 128 index cases were enrolled in this study. This group comprised 72 HBOC (58 breast cancer cases and 14 ovarian cancer cases) and 57 HNPCC, positive for the Amsterdam criteria. They were selected by the Regional Hereditary Cancer Prevention Program of Castile & Leon (Spain). Ethical committee approval, informed consent, family history and clinical features were collected.

### DNA and RNA extractions

Genomic DNA from peripheral blood was automatically extracted by Roche MagNaPure^®^ Compact, using the “MagNA Pure Compact Nucleic Acid Isolation Kit I—Large Volume” (Roche Diagnostics, Penzberg, Germany), following the manufacturer’s instructions. RNA was extracted from peripheral blood lymphocytes using the GeneMATRIX Human Blood RNA Purification Kit (EURx, Gdánsk, Poland).

### Mutation screening

All the DNA samples were screened for germline mutations using the On-Demand Research Assay on the Ion S5 system (both ThermoFisher Scientific, Waltham, MA, USA). Each DNA sample was checked for concentration using a Qubit 3.0 Fluorometer (Thermo Fisher Scientific). The concentration of input DNA was then adjusted to 50 pmol.

The library and template preparations were performed using the automated Ion Chef System, then sequenced in Ion S5 with Ion 520 Chip (all Thermo Fisher Scientific) according to the manufacturer’s instructions.

Sequencing results were aligned to the hg19 human reference genome and analyzed using the Ion Reporter Software Version 5.10 (Thermo Fisher Scientific).

The variant frequency cut-off for the detection of gene germline mutation was defined as 20%, as recommended by a previous study [7].

The On-Demand Research Assay showed 99.85% sensitivity, 100% specificity, 0% false-positive rate and 99.99% accuracy in detecting single nucleotide variations (SNVs) and small deletions.

### Sanger sequencing

Direct automated Sanger sequencing was used to confirm the results detected by massive parallel sequencing. For this purpose, we used the BigDye Terminator Sequencing Kit v3.1 (Applied Biosystems) on an ABI 3100 DNA Sequencer (four capillaries; Applied Biosystems). Co-segregation studies were conducted when possible.

### RT‐PCR

When Human Splicing Finder (HSF) software (http://www.umd.be/HSF3/) predicted a splicing disruption caused by a mutation, we performed a cDNA-based analysis. To this end, RNA isolated from lymphocytes was reverse transcribed into cDNA using the Transcriptor First Strand cDNA Synthesis Kit (Roche), according to the manufacturer’s protocol. We then evaluated the possible impact on transcription by performing a PCR, subsequently separating the products in low melting 2% agarose gel with Red Safe™ staining. After that, the bands were isolated and the DNA extracted using NucleoSpin^®^ Gel and PCR Clean‐up (Macherey‐Nagel, Düren, Germany) was sequenced. Oligonucleotides and PCR conditions are available on request.

### In-silico analyses

Mutations with protein annotations and minor allele frequency (MAF) < 0.01, according to ExAC data, were analyzed using the Cancer Genome Interpreter (https://www.cancergenomeinterpreter.org) and the Human Splicing Finder TM 3.0 (HSF) and (http://www.umd.be/HSF3). To assess the potential repercussion of the variants on the protein functionality, we used the Combined annotation-dependent depletion (CADD) method which releases scaled C-scores related to the top ranked pathogenicity: CADD-Score-10 means you are in the top 10% of the disrupting mutations, CADD-Score-20, top 1%, CADD-Score-25, top 0.5% and CADD-Score-30, 0.1%. CADD integrates diverse annotations into a single score by contrasting variants that survived natural selection with simulated mutations [8].

### Variant classification

Variants were classified as deleterious if: they originated a premature stop codon, they were located in canonical splice sites, or there was literature evidence. The potential deleteriousness of the remaining variants was evaluated using the Combined Annotation-dependent Depletion (CADD) method. We considered variants with a CADD-score of > 20 as interesting to be considered (top 1% of disrupting variants).

### Genotype–phenotype correlations and statistical analysis

Genotype-phenotype correlations between personal/familial data and mutation profiling of the samples were examined. Chi square tests were used to investigate the relationships between the categorical variables and Spearman’s Rank correlation coefficient was used to assess the strength of association between two variables. Statistical tests were carried out using the R statistical programming language (v3.5.1).

## Results

We have integrated an On-Demand Panel comprising 35 genes associated with cancer predisposition syndromes, in particular HBOC and HNPCC, into our routine genetic testing (Table 1). All the genes have been previously identified as cancer predisposition genes. The analysis with the On-Demand panel has simplified and standardized the laboratory workflow in a single procedure to test hereditary syndromes. The 128 samples included in this study were sequenced in five consecutive experiments.

**Table 1:** Genes included in the customized On-Demand Panel

Penetrance	Syndrome	Gene (reference sequence)
High	HBOC	BRCA1 (NM_007300.3) BRCA2(NM_000059.3)
HIgh	HNPCC	MLH1 (NM_000249.3) MSH2 (NM_000251.2) MSH6 (NM_000179.2) PMS2 (NM_000535.6) EPCAM (NM_002354.2)
High	Others	APC (NM_000038.5) BMPR1A (NM_004329.2) CDH1 (NM_004360.4) CDK4 (NM_000075.2) MUTYH (NM_005591.3) KRAS (NM_033360.3) PTEN (NM_000314.4) SMAD4 (NM_005359.5) STK11 (NM_000455.4) TP53 (NM_000546.5)
Moderate-Low	Multiples	ATM (NM_000051.3) ATR (NM_001184.3) BLM (NM_000057.3) BARD1 (NM_000465.3) BRIP1 (NM_032043.2) CHEK2 (NM_007194.3) FAM175A (NM_139076.2) NBN (NM_002485.4) MEN1 (NM_000244.3) PALB2 (NM_024675.3) FANCM(NM_020937.3) MRE11A(NM_005591.3) PRKAR1A (NM_212471.2) RAD50 (NM_005732.3) RAD51C (NM_058216.2) RAD51D (NM_001142571.1) POLD1 (NM_001256849.1) POLE (NM_006231.3)

*HBOC* Hereditary Breast and Ovarian Cancer, *HNPCC* Hereditary Non Polyposis Colon Cancer

Coverage uniformity was higher than 90% in all tested samples. The average value of total aligned reads was 1,040,207 (89%), and the average percentage of target coverage at 50 × was 88.6%, the median region coverage depth being 206× (range: 29–549).

The sequencing results were then filtered during the bioinformatics analysis and only selected variants that met the quality criteria were analyzed. Sufficient coverage was sought to ensure that all bases within ROIs were covered at a minimum of 30×. The Ion reporter pipeline parameters were adjusted to ensure greater control over the variant calling quality. In a first approach, a training set of different mutations in high penetrance genes was used to evaluate the performance of the panel. All the variants were both correctly sequenced and annotated (data not shown).

A total of 18 Pathogenic or Likely Pathogenic variants (PV/LPV) were identified in 18 cancer cases (14%), affecting 9 different genes with a current clinical utility for each hereditary cancer condition (Table 2).

**Table 2:** Pathogenic variants (PV)

Syndrome	Cancer type	Gene	cDNA	Protein	Consequence
HNPCC	Ov	*ATM*	c.5979_5983delTAAAG	p.Ser1993Argfs	Frameshift
HNPCC	Ov	*ATM*	c.1339C > T	p.Arg447Ter	Nonsense
HBOC	Br	*BLM*	c.1642C > T	p.Gln548Ter	Nonsense
HBOC	Br	*BRCA2*	c.658_659delGT	p.Val220fs	Frameshift
HBOC	Br	*BRCA2*	c.2808_2811delACAA	p.Ala938fs	Frameshift
HBOC	Br	*BRIP1*	c.206-2A > G	–	Splicng
HNPCC	Ov	*BRIP1*	c.1140 + 1G > C^a^	–	Splicng
HBOC	End	*CHEK2*	c.593-1G > T	–	Splicng
HBOC	Col	*CHEK2*	c.1427C > T	p.Thr476Met	Missense
HNPCC	Gas	*MLH1*	c.2239_2240insAGCCTGATACTATATCCTGCAGC	p.Pro747fs	Frameshift
HNPCC	Br	*MSH2*	c.211G > C	p.Gly71Arg	Missense
HNPCC	Br	*MSH2*	c.2131C > T	p.Arg711Ter	Nonsense
HNPCC	Br	*MUTYH*	c.1147delC	p.Ala385fs	Frameshift
HNPCC	Skin	*MUTYH*	c.1187G > A	p.Gly396Asp	Missense
HNPCC	Col	*MUTYH*	c.1187G > A	p.Gly396Asp	Missense
HNPCC	Gas	*MUTYH*	c.1187G > A	p.Gly396Asp	Missense
HNPCC	Col	*MUTYH*	c.1187G > A	p.Gly396Asp	Missense
HNPCC	Col	*PMS2*	c.137G > T	p.Ser46Ile	Missense

cDNA and Protein changes are named according to the HGVS nomenclature

Reference sequence: ATM (NM_000051.3), BLM (NM_000057.3) BRCA1 (NM_007300.3) BRCA2(NM_000059.3) BRIP1 (NM_032043.2) CHEK2 (NM_007194.3) MLH1 (NM_000249.3) MSH2 (NM_000251.2) MSH6 (NM_000179.2) MUTYH (NM_005591.3) PMS2 (NM_000535.6)

The cancer type of the index case is indicated according to the following abbreviations: *Br* breast cancer, *Col* colon cancer, *End* endometrial cancer, *Gas* gastric cancer, *Ov* ovarian cancer, *Skin* skin cancer

^a^Not previously reported

These alterations represented 13 Single Nucleotide Variants (SNVs), 4 deletions and 1 insertion, all in heterozygosis, and resulted in: 7 missense variants, 5 frameshift variants, 3 nonsense variants (resulting in premature termination codon) and 3 splicing variants (1 not yet reported in consulted databases). This group with PV comprised 6 HBOC and 12 HNPCC individuals fulfilling the Amsterdam criteria.

Focusing on affected genes, the most frequently mutated gene was *MUTYH* with 5 variants, 4 being the same monoallelic mutation p.Gly396Asp. Interestingly, the MAF for this mutation in ExAc is very low in comparison to ours, suggesting a high frequency in our population. Regarding the affected carriers, breast, ovarian, colon and stomach were the cancer types. The other *MUTYH* PV was p.Ala385fs in a gastric cancer case. For *ATM*, *BRCA2, BRIP1, CHEK2* and *MSH2*, two PVs were found in each gene. Finally, one PV was identified in the following genes: *BLM, MLH1* and *PMS2*. Both ATM PV (p.Ser1993Argfs and p.Arg447Ter) and one of the BRIP1 mutations (c.1140 + 1G > C) were found in ovarian cancer cases; BRCA2 (p.Val220fs and p.Ala938fs), CHEK2 (c.593-1G > T and p.Thr476Met), BLM (p.Gln548Ter) and the other BRIP1 PV (c.206-2A > G) were identified in women affected by breast cancer. Interestingly, the p.Pro747fs PV in MLH1 was detected in a breast cancer patient and the p.Gly71Arg in MSH2 in a woman diagnosed with skin cancer. Conversely, as expected, the other MSH2 PV (p.Arg711Ter) and the PMS2 mutation, p.Ser46Ile, appeared in colon cancer cases. It is worth noting that the ovarian cancer patients harbouring ATM PV, as well as the breast cancer cases with CHEK2 PV, present a familial history matching both HNPCC and HBOC criteria.

**Fig. 1:**
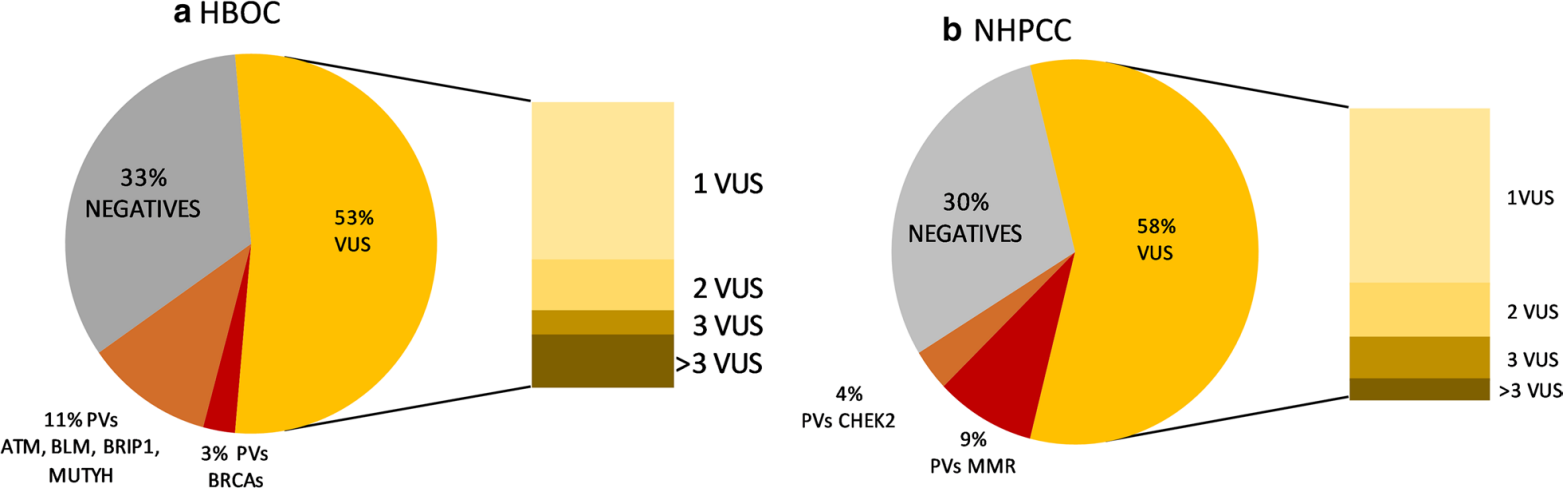
Representation of the different percentages of patients defined by the different types of mutation according to the Hereditary Cancer Syndrome. **a** For HBOC, the mutation rates for patients with PV in *BRCA1* and *BRCA2* genes (3%) was significantly lower than the 11% represented by PV in *ATM, BL*M, *BRIP1* and *MUTYH* genes, which were not screened routinely for HBOC. A total of 53 of the cases carried 1, 2, 3 or more (up to 6) VUS. In the 33% of the analyzed samples, we did not find any relevant variant (negatives); **b** For HNPCC, the mutation rates for patients with PV in the MMR genes (9%) was significantly higher than the PV identified in other different genes, which in this case only refers to *CHEK2*. A total of 58% of the cases carried 1, 2, 3 or more (up to 5) VUS. In 30% of the analyzed samples, we did not find any relevant variant

One of the questions raised is whether, by applying an On-Demand panel, we enhanced the testing potential as compared with screening BRCA and MMR genes exclusively. For this question, we estimated the productivity of the panel design, comparing the diagnostic yields of PV found in traditionally screened genes and others. Using this comparative analysis, we aimed to determine the increase in PV due to the incorporation of other genes different from those traditionally analyzed (BRCA genes and MMR genes). Furthermore, to assess the suitability of the panel according to the type of hereditary cancer syndrome, we performed the analysis for each syndrome, HBOC and HNPCC. The results are shown in Fig. 1.

In the case of HBOC, the PV found in genes other than BRCA1 and BRCA2 represented 11% versus the 3% corresponding to BRCA genes (in particular, all the PV were found only in BRCA2). Conversely, for HNPCC, the majority of PV still resided in the traditionally screened genes, representing 9% versus the 4% with other genes, in particular, *CHEK2*. If we only pay attention to PVs with a clinical applicability, the designed On-Demand panel was much more efficient in the case of HBOC than HNPCC. This would pose the premise of not using the same customized panel for the two different syndromes and, hence, to consider that different designs were necessary.

As expected, the 35 genes panel implied the detection of a large range of VUS, representing more than 50% of the genetic results for both syndromes. In the case of HBOC, 29% of cases carried one VUS; 10% of cases carried 2; 4% of cases 3 and 10% of cases more than 3 VUS. For HNPCC, 35% of cases carried one VUS; 11% of cases 2; 9% of cases 3 and 4% of cases more than 3 VUS.

In the clinics, VUS were not representing an added value, yet, nonetheless, these variants could offer an alternative explanation for cancer genetic predisposition based on the polygenic model. Besides, the large percentage represented by VUS indirectly reduced the total number of negative cases in both syndromes, HBOC and HNPCC.

**Fig. 2:**
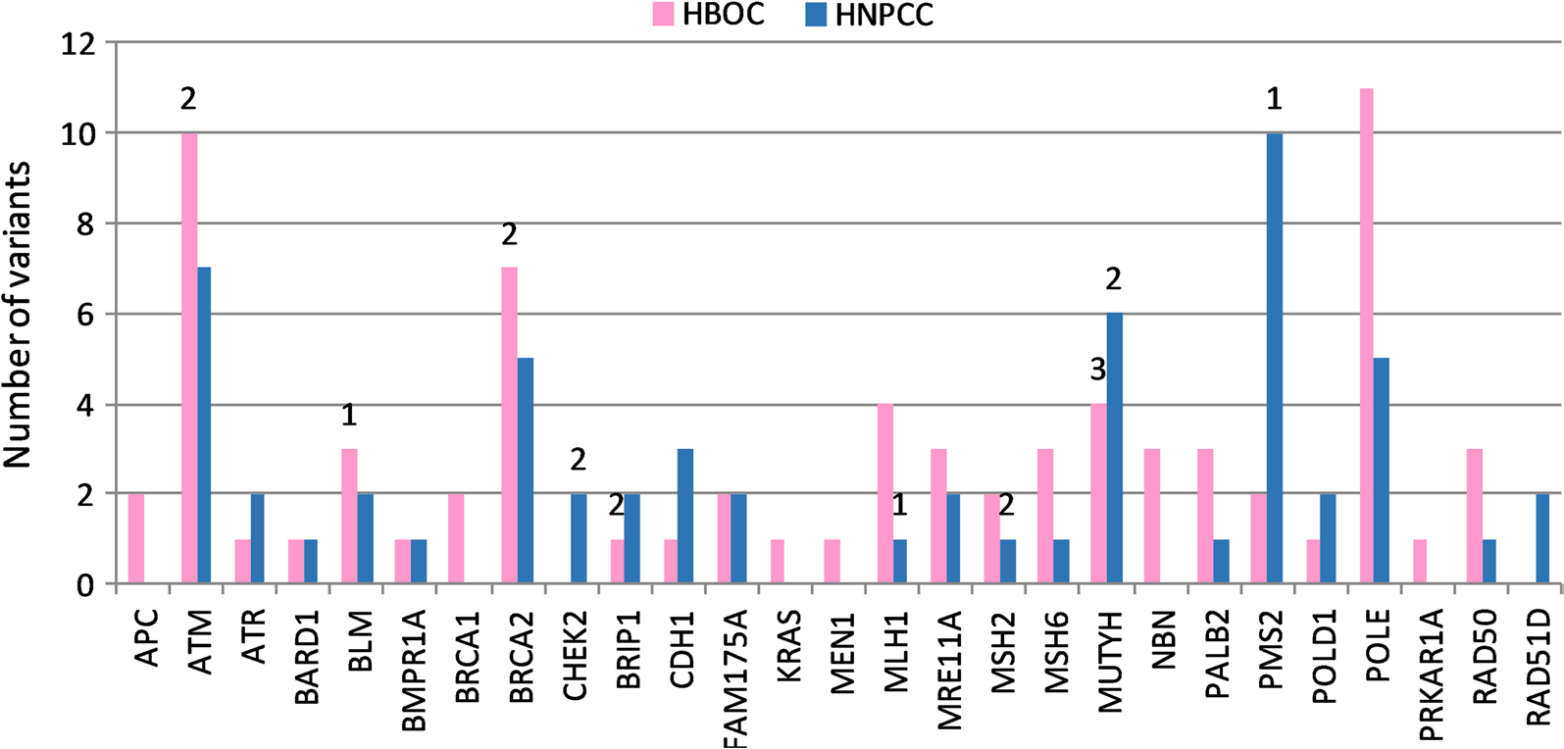
Distribution of the PV and VUS along the different genes according to the Hereditary Cancer Syndrome. The bar diagram represents the number of variants identified in the different genes. The bars define the number of VUS detected: pink bars correspond to HBOC and blue bars to HNPCC. The number of PVs is indicated in the upper part of the bar for the respective gene

We depicted the distribution of the PV and VUS along the different genes and for each syndrome. In the case of HBOC, a total of 71 relevant variants were identified; for HNPCC, 60 variants. In Fig. 2, it can be appreciated that *ATM, BRCA2, MUTYH, POLE* and *FAM175A* were frequently mutated, accumulating 58% of the variants in HBOC and 45% in HNPCC; for other genes, variant distribution was divergent. In particular, in a number of genes for HNPCC, a non-relevant variant was found, compromising their utility in our case cohort. Interestingly, several VUS and PV were identified in *MUTYH* in HBOC, a gene normally studied exclusively in the context of HNPCC.

To take advantage of the results from the NGS implementation in our diagnostic routine, we further investigated the VUS with MAF < 0.01, performing an in silico analysis using CADD. A CADD score > 20 is indicative of possible functional repercussions caused by the variant (Additional file 1: Table S1).

**Fig. 3:**
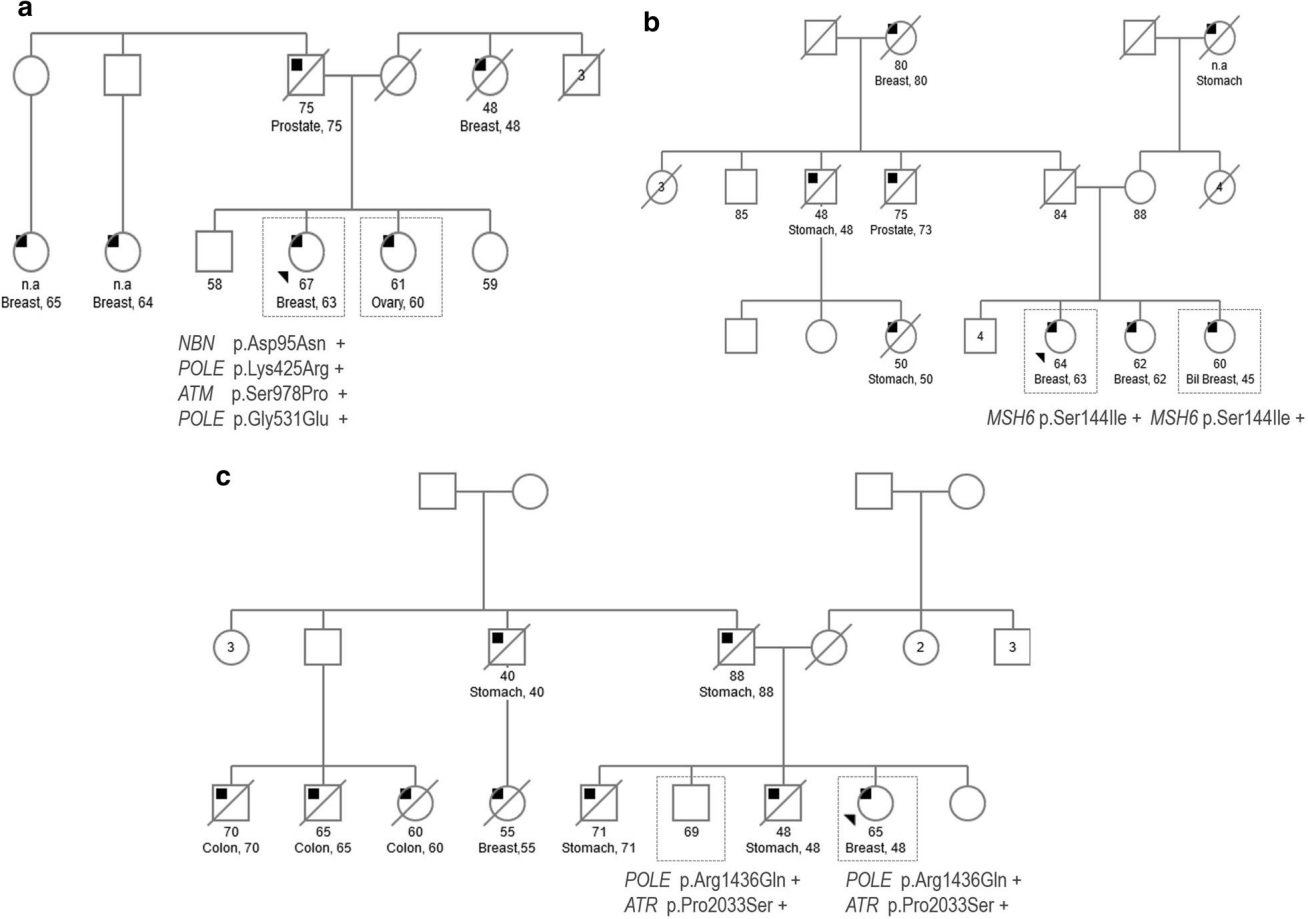
Segregation analysis of VUS. The segregation of some VUS was evaluated in three different families. The pedigrees show the different types of tumor in the affected probands, represented with an inner black square, and the diagnosis and current age when available. The index case is marked with an arrowhead. The individuals tested for the indicated mutations are surrounded by a square. Positive results are represented by “+” and negative ones by “−”

We were able to analyze the segregation of VUS in a limited number of individuals, from 3 families, to add more information concerning their possible role in the phenotype through an accumulative effect (Fig. 3). In the case of family A, four variants with conflicting interpretations about pathogenicity were detected in the index case, a woman diagnosed with breast cancer at the age of 62. Two out of the four variants (*NBN* p.Asp95Asn and *POLE* p.Lys425Arg) were also detected in her sister, who developed ovarian cancer. In family B, the VUS in *MSH6* p.Ser144Ile segregated with the two analyzed cases of breast cancer, one of them being bilateral, a hallmark of hereditary cancer. In family C, as the different cancer cases were deceased, we could only test a healthy brother of the index case who developed breast cancer for the VUS (p.Arg1436Gln in *POLE* and p.Pro2033Ser in *ATR*); the two of them carried both variants.

**Fig. 4:**
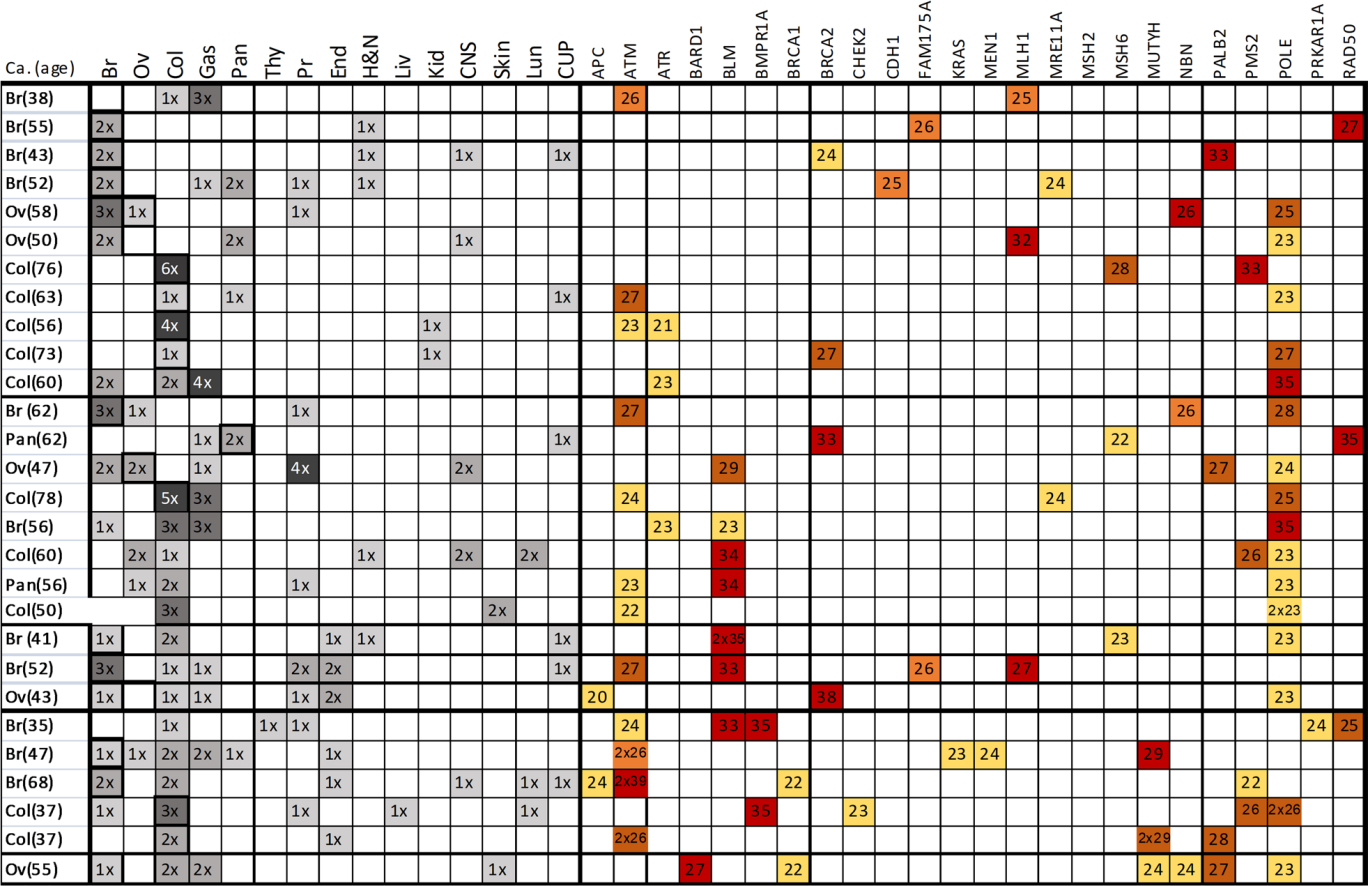
Correlation between cancer history and genetic results for high CADD score variants. Representation of the personal and family history of the samples carrying two or more VUS with high CADD scores. For each variant, the CADD-score and the gene affected is indicated. The different tumor types for the index case and the family members are coded as following: Br, breast; Ov, ovarian; Col, colon; Pan, pancreas; Gas, gastric; Thy, thyroid; Pr, porstatic; End, endometrial; H&N, head and neck; Liv, liver; Kid, kidney; CNS, central nervous system; Skin, skin; Lun, lung and CUP, cancer of unknown primary origin. When multiple cancer cases in the family or several variants in the same gene existed, we used the “x” symbol to represent the multiplicative condition: 1x (1), 2x (2), 3x (3), etc

Intriguingly, some patients accumulated several high-CADD-score variants: 11 cases with 2 VUS, 8 cases with 3, 3 cases with 4, 5 cases with 5 and one case carried a total of 6 VUS. These rare missense variants with predicted functional implications would have a summatory effect, according to the polygenic risk model. It is worth noting that, by inspecting the family cancer histories, we observed that index cases carrying several VUS belonged to families with high tumor type diversity. Figure 4 compiles the family history of the studied cohort and the information about the respective VUS, both the CADD score and the gene in which were found.

**Fig. 5:**
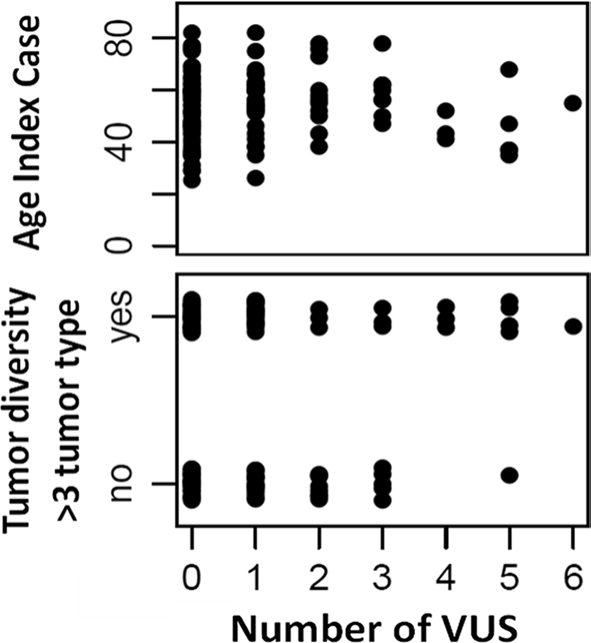
Association between the age of diagnoses of the index cases and tumor diversity in the respective families with the number of VUS identified in the sample. The upper plot shows how the condition of carrying 3 or more VUS could be determining early cancer onset. The lower plot represents the possible association of the categorical variable “tumor diversity in the family” defined as ‘yes’ when the pedigree harbored more than 3 tumor types. In particular, for the condition of carrying 4, 5 and 6 VUS, the tumor diversity in the pedigrees tends to increase

We explored a possible correlation between the genetic condition of carrying VUS (ranging from 1 to 6) and the ages of diagnosis of the index cases. There was a weak negative correlation between the genetic condition of carrying VUS (ranging from 1 to 6) and the ages of diagnosis of the index cases (Spearman rho = − 0.376; Fig. 5). Next, we tested whether there was an association between the condition of carrying VUS and the tumor diversity in the family. Considering the two binary variables: “carrying two or more VUS” and “having three or more different tumor types inside a family”, the association was not statistically significant (Chi square test, p = 0.348). Nevertheless, when the first variable was re-defined as ”carrying three or more VUS”, the association was significant (Chi square test; p = 0.045).

When visualizing the data, although it was not statistically significant, we did observe a trend: cases carrying a higher number of VUS (starting from 4 VUS onwards) and belonging to a family harbouring more than 3 different cancer types (higher tumor diversity), presented an earlier onset of cancer (Fig. 5).

**Fig. 6:**
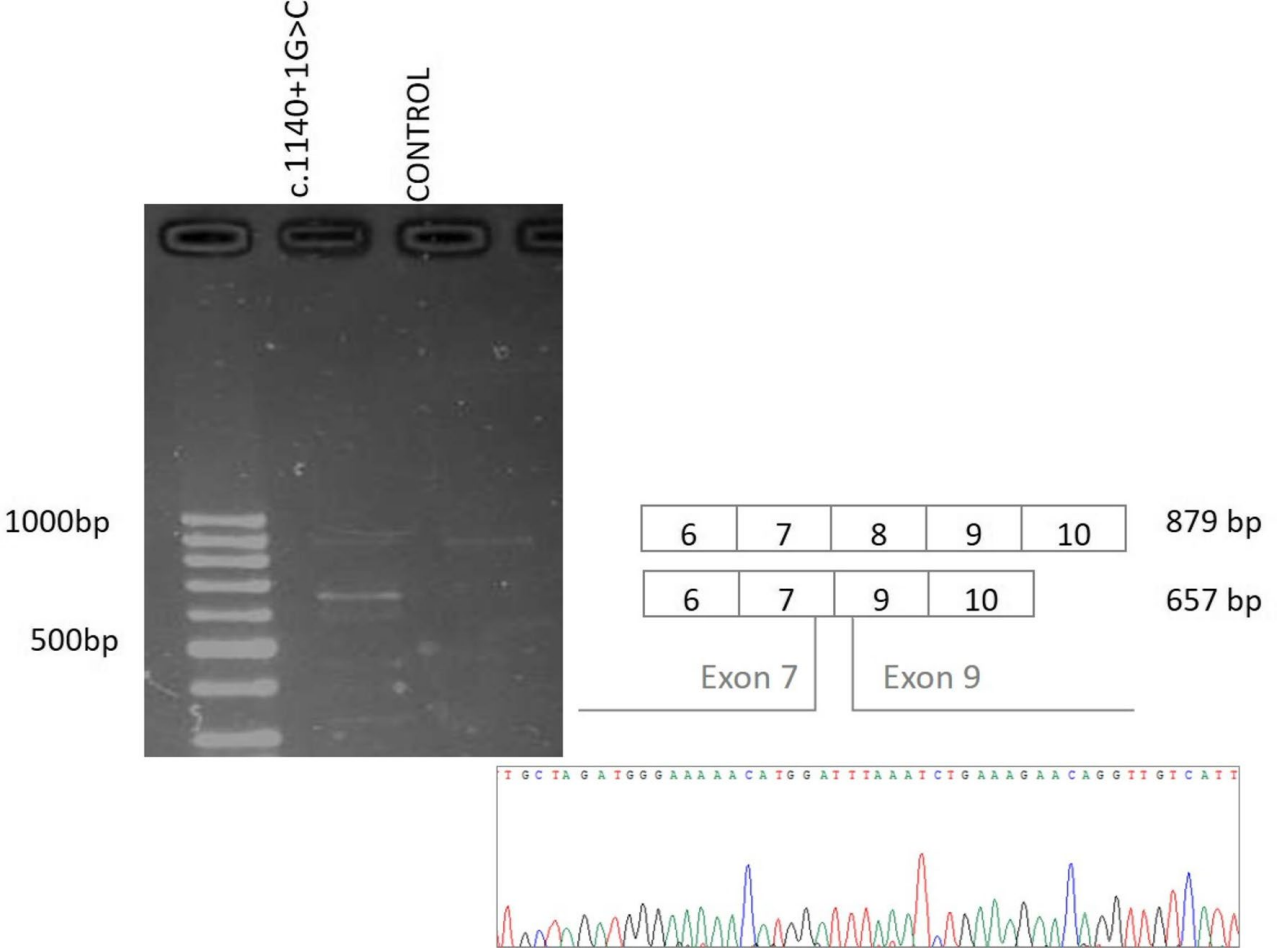
Characterization of the c.1140 + 1G > C variant in *BRIP1* at RNA level. The amplified PCR products were separated by a 2% agarose gel electrophoresis to detect the possible aberrant transcripts. Then, Sanger sequencing confirmed the different exon exclusions. The c.1140 + 1G > C variant in *BRIP1* caused the skipping of exon 8, resulting in a product which is 222 nucleotides shorter than the full-length product (879 nucleotides)

Since splicing disruption can lead to dysfunctional protein products, we studied, at RNA level, those mutations that affected canonical or regulatory splicing sites. Regarding mutations located in canonical splicing sites, we confirmed the splicing disruption caused by a not previously reported c.1140 + 1G > C *BRIP1* mutation. In Fig. 6, we can observe the presence of aberrant transcripts for the carrier of the c.1140 + 1G > C variant in *BRIP1*; while the control cDNA only presented one band that corresponded to the wildtype transcript and the carrier cDNA showed an extra band in the agarose gel. Sequencing of the aberrant transcript subsequently confirmed an exon 8 skipping for the carrier of the c.1140 + 1G > C *BRIP1* variant.

In addition, HSF indicated that the c.4076 + 4T > G variant in *BLM*, and c.4473C > T in *ATM*, could potentially alter the splicing. Moreover, the literature presented conflicting interpretations of pathogenicity for both variants. Specifically, the c.4076 + 4T > G variant in *BLM* could activate an intronic cryptic donor critical site, a new donor site. On the contrary, c.4473C > T in *ATM* could result in the breaking of an exonic enhancer site. We did not detect any aberrant transcript, either for the c.4076 + 4T > G variant in *BLM* or for c.4473C > T in *ATM.*

In an attempt to contribute to clinical actionability, in particular to therapy selection, we consulted the OncoKB database. We were looking for a registered response to specific targeted drugs in relation to the mutation profile of the patients. It may be considered that the *ATM, BRCA2* and *CHEK2* pathogenic mutations could be determining a PARP inhibitor sensitivity, as already registered. Moreover, in the case of *ATM* pathogenic mutations, the DNA-PKc and ATR inhibitors were expected to be effective. According to this information, a total of 6 patients could benefit from targeted therapy options.

## Discussion

To apply successful clinical management in the context of hereditary cancer, defining the cause of the inherited predisposition is crucial. Mutations in traditionally screened, high penetrance genes explain a small number of cases [9]; nevertheless, new strategies are needed to explain the predisposition that remains unsolved.

Next Generation Sequencing, in particular multi-gene panel application, has proved to be successful in the context of Hereditary Cancer diagnoses [4, 10, 11]. The implementation of the NGS in our laboratory has confirmed the technique’s cost-effectiveness: the On-Demand panel allowed us to analyze a total of 128 samples in 4 consecutive experiments (5 days per experiment, including bioinformatic analysis), corroborating the scalability and reducing the turnaround time enormously [12]. In addition, using targeted sequencing, we ensured enough coverage in the regions of interest, guaranteeing a robust variant calling [13].

In our study, more than 50% of the samples carried one or more VUS, followed by more than 30% in non-informative cases and 14% with a PV, rates similar to other groups using NGS multi-gene panels [4, 14].

Focusing on PV, the genetic screening has a direct impact on the patients; once the genetic risk is identified, preventive measures and management could be offered [15]. Although the clinical actionability is limited by the reduced number of guidelines [16, 17], genetic counselling is possible for most of the PVs identified [18, 19].

One of the questions raised is whether, by applying the On-Demand panel, we enhanced the testing potential not only detecting pathogenic mutations in BRCA and MMR genes, but also in other genes not traditionally screened. In that regard, the On-Demand Panel allowed us to detect PV in 10 cases that had gone unnoticed with the traditional screening of the classical high penetrance BRCAs and MMR genes. In particular, this increased ratio of PV due to the On-Demand panel was more evident for HBOC (11%), as compared to the modest increased yield (3%) of the extended analysis in HNPCC. This poses the premise that a particular design, based on the clinical phenotype of the cases, would be needed. In this regard, laboratories differed in their opinions of whether phenotypic overlap warrants offering pan-cancer panels only versus cancer specific panels [20]. One conservative but convenient strategy would seem to be the use of phenotype-driven panels with opportunistic testing of the traditional, high penetrance BRCA and MMR genes [6]. In spite of the optimizations that need to be carried out, the On-Demand panel application led to reports of mutations in moderate penetrance genes that would otherwise have gone undetected [16].

Gene panel testing implies the identification of VUS [16, 21]. While the classification of VUS is a particular limitation and challenge in clinical applicability [22]; in a research context, these VUS can be further investigated to explore the genotype–phenotype correlation and to explain the genetic predisposition using the polygenic model. On the one hand, restrictive filters and in silico tools may be used to focus attention on a reduced list of mutations. On the other hand, the coexistence of several VUS in a number of patients would give us the opportunity to explain the genetic causality using the polygenic model; the accumulative functional repercussions of several VUS may be conferring a significant risk. Other authors have associated co-occurring variants in DNA repair genes with an earlier onset of breast cancer, suggesting a summation effect [23]. In our study, the global analysis revealed a complex variation landscape, where the number of variants with a possible functional impact per individual ranges from 1 to 6. Similarly to other analyses [24], our data reveals a diversity in the number of variants detected in the different genes, but only 5 out of the 35 genes accumulated 50% of the VUS and PVs.

As the in silico analysis cannot resolve the clinical significance of a variant [25], we performed segregation studies on a limited number of families. The mutations p.Asp95Asn in *NBN* and p.Lys425Arg in *POLE* segregated with both breast and ovarian cancer cases (Fig. 3a); the VUS in *MSH6* p.Ser144Ile was identified in the two cases of breast cancer, one of them bilateral, in an HNPCC family (Fig. 3b). Although the variant had previously been identified in colon cancer cases [26], our analysis poses the possible contribution to the breast cancer phenotype. In a family that has colon, gastric and breast tumors, the variants p.Arg1436Gln in *POLE* and p.Pro2033Ser in *ATR* were identified in a breast cancer case and in her healthy brother of the index case. Variants affecting the functionality of ATR have been related with high risk for colon [27] and breast cancer [28]. Regarding mutatons in *POLE,* their contribution to colorectal cancer predisposition has been described [29]. Moreover, the p.Arg1436Gln variant in *POLE* presented a very high-CADD-score, 35. Therefore, taking into account the family history, it would be worth programming control endoscopies on the unaffected individual carrier. On the other hand, the identified VUS in this family, and the coexistence of colon, gastric and breast cancer cases, highlight the overlapping phenotypes between the different cancer phenotypes. This makes the targeted molecular testing for single genes less favorable. Along these lines, expanding the spectrum of the studied genes by panel testing could bring unexpected genotype- cancer phenotype correlations.

As previously outlined, although family segregation studies can yield powerful data to reclassify a VUS, the small size of the family and the very limited number of recruited probans limited its potential [30]. Nevertheless, family co-segregation studies should be considered in the clinical routine to add more information to the genetic findings.

Although no significant genotype–phenotype associations were found, some notable observations can be pointed out: the condition of carrying several VUS (especially more than 3) could be determining a younger diagnosis age in the cases and higher tumor diversity in their respective families. The analysis of large cohorts could allow us to establish these putative correlations.

We identified a new splicing *BRIP1* variant, c.1140 + 1G > C. In Clinvar, there is a change of G > A in the same position, which classifies it as probably pathogenic. It is known that variants affecting splicing donors or acceptor sites imply a loss of protein function [31] and this loss of function of BRIP1 has a pathogenic effect [32]. To get an insight into the potential effect of this variant, we performed a characterization at RNA level, confirming the fact that the nucleotide change, located in a splicing donor site, caused a loss of exon 8.

Splicing alterations are normally the result of mutations in the intronic flanking regions of the exon, but some exonic variants can affect mRNA processing, triggering a functional alteration [33]. The mechanism is probably based on the alteration of potentiating sites (ESE) or splicing silencers (ESS) located in exonic regions [34]. Therefore, we explored the possible impact on the mRNA processing of two exonic variants predicted by HSF as possible splicing disruptors: the c.4076 + 4T > G variant in *BLM* and c.4473C > T in *ATM*. Neither the *BLM* mutation nor the *ATM* variant caused an aberrant RNA processing in the patient samples. Consequently, although the HSF algorithm has proven to be an efficient computational tool for predicting splicing alterations [35], whenever possible, an effort should be made to confirm the prediction at RNA patient level.

As previously remarked, the comprehensive gene panel was created to potentiate the detection of PVs. Intriguingly; some PVs are clustered in genes not directly related to the initial established phenotype according to family history. In light of this, two major observations must be highlighted. First, the monoallelic PV in *MUTYH* c.1187G > A (p.Gly396Asp) was identified in four unrelated patients, a significantly high frequency when compared to the data registered in ExAC (0.002). Given the fact that *MUTYH* is a recessive gene [36], *MUTYH*-monallelic-PV could not explain the cancer phenotype; in contrast, several recent studies support the possible role of monoallelic mutations in *MUTYH* conferring risk [37] for colorectal, breast, gastric and endometrial cancer. In particular, the patients with the c.1187G > A variant in *MUTYH* were diagnosed with ovarian, endometrial, colorectal and gastric cancer, respectively, emphasising the possible contribution of PV in *MUTYH* to these cancer types, even heterozygous. Second, we identified two deleterious alterations in *CHEK2, c.593*-*1G *> *T* and *p.Thr476Met,* in two patients who developed breast cancer, a cancer type related to PV in this gene [38]. Intriguingly, the individual phenotype of both patients, breast cancer (in contrast to the familial phenotype, HNPCC), would be determining different preferences regarding the list of genes to screen. However, in this situation, there is an overlap of cancer types that may prompt us to take into account mutations in genes not exclusively correlated with the familial syndrome. Conversely, the patient with the p.Pro747fs variant in *MLH1* developed breast cancer at the age of 47. In principle, *MLH1* would not be a candidate gene in breast cancer screening but, in this case, the fact that her family matched criteria for HNPCC allowed us to determine the PV in the patient.

Therefore, screening genes corresponding to other cancer phenotypes could give us the opportunity to define the testing genetic profile, especially when the family history gathers several cancer types, matching more than one hereditary cancer syndrome.

In the new era of targeted therapy in cancer, drug-response records may serve as a reference for treatment selection. The fact that many of the proposed treatments are based on the synthetic lethality principle, in combination with the mutation profile, would lead to increased effectiveness and a decrease in side effects. Knowing that the transcendence of the genetic test results depends on its transference to clinical practice, we wanted to use the genetic information for treatment selection, making the application of the concept of personalized medicine plausible [39, 40]. According to other studies, the PV in *ATM, BRCA2* and *CHEK2* could be determining a PARP inhibitor sensitivity [41]. In addition, for *ATM* PV, a synthetic lethality synergism has been described with DNA-PKc and ATR inhibitors [42]. This information could be considered to design targeted therapy options in 6 of our patients.

## Conclusion

On the basis of the preceding discussion, the implementation of gene-panels can improve the clinical management of affected families in a quick and cost-effective method. Moreover, extending the analysis to other genes renders the opportunity to discover infrequent alterations and to provide a reliable molecular portrait of different cancers.

## Supplementary information

**Additional file 1.Table S1.** CADD score of the different VUS identified in the genetic analysis.

## Data Availability

All data are available upon request.

## Abbreviations

CADD: Combined annotation-dependent depletion
HBOC: Hereditary Breast and Ovarian Cancer
HNPCC: Hereditary Nonpolyposis Colorectal Cancer
HSF: Human Splicing Finder
MAF: Minor Allele Frequency
NGS: Next Generation Sequencing
PV/LPV: Pathogenic or Likely Pathogenic variants
SNVs: Single Nucleotide Variants
VUS: Variants of Unknown Significance
